# In situ electrosynthetic bacterial growth using electricity generated by a deep-sea hydrothermal vent

**DOI:** 10.1038/s41396-022-01316-6

**Published:** 2022-09-23

**Authors:** Masahiro Yamamoto, Yoshihiro Takaki, Hiroyuki Kashima, Miwako Tsuda, Akiko Tanizaki, Ryuhei Nakamura, Ken Takai

**Affiliations:** 1 Institute for Extra-cutting-edge Science and Technology Avant-garde Research (X-star), Japan Agency for Marine-Earth Science and Technology (JAMSTEC), Yokosuka, Japan; 2 Center for Sustainable Resource Science, RIKEN, Wako, Japan; 3 Earth-Life Science Institute (ELSI), Tokyo Institute of Technology, Tokyo, Japan

**Keywords:** Soil microbiology, Biogeochemistry

## Abstract

Electroautotrophic microorganisms have attracted great attention since they exhibit a new type of primary production. Here, in situ electrochemical cultivation was conducted using the naturally occurring electromotive forces at a deep-sea hydrothermal vent. The voltage and current generation originating from the resulting microbial activity was observed for 12 days of deployment, with fluctuation in response to tidal cycles. A novel bacterium belonging to the genus *Thiomicrorhabdus* dominated the microbial community specifically enriched on the cathode. Metagenomic analysis provided the draft genome of the bacterium and the gene repertoire indicated that the bacterium has the potential for thio-autotrophic growth, which is a typical physiological feature of the members of the genus, while the bacterium had a unique gene cluster encoding multi-heme cytochrome *c* proteins responsible for extracellular electron transfer. Herein, we propose this bacterium as a new species, specifically enriched during electricity generation, as ‘*Candidatus* Thiomicrorhabdus electrophagus’. This finding suggests the natural occurrence of electrosynthetic microbial populations using the geoelectricity in deep-sea hydrothermal environments.

## Introduction

Electroactive microorganisms (EAMs), which can directly receive and/or release electrons from/to extracellular solid materials, have been of interest for several decades [1–5]. *Geobacter sulfurreducens* and *Shewanella oneidensis* MR-1 are well-known model EAMs, although many different microorganisms, including bacteria, archaea and eukaryotes, can exchange electrons with electrodes [4, 6, 7]. EAMs have been observed in various environments, such as wastewater, rice paddy soil, seawater, and marine sediment [8]. EAMs that release electrons to extracellular electrodes as terminal electron acceptors are called “electrogens”, while those that accept electrons from extracellular electrodes as electron donors are called “electrotrophs” [4]. In addition, the autotrophic growth of electrotrophs utilising carbon dioxide as a carbon source is called “electrosynthesis”. This term is used in engineering to describe useful chemical production, whereas it is used in biochemistry as the counterpart of photosynthesis and chemosynthesis, and the concept is very important because electricity can be an alternative energy source for biomass production instead of light and oxidoreduction reactions between inorganic chemicals.

Several isolated and enriched autotrophs such as *Rhodopseudomonas palustris*, *Acidithiobacillus ferrooxidans*, and ‘*Candidatus* Tenderia electrophaga’, showed electron uptake and CO_2_ fixation in electrochemical cultivation systems in the laboratory, although their growth rates and biomass production were quite limited [9–11]. These electrosynthetic bacteria possess different mechanisms for the extracellular electron uptake (EEU) pathway, indicating different evolutionary traits [9, 10, 12, 13].

In various natural environments, a sufficient amount of electricity can be supplied via a redox gradient, and an electromotive force and conductive materials, such as biogenic filaments and minerals, can fuel potential electrosynthetic microbial populations [14, 15]. However, it has been difficult to specifically detect the electrosynthetic production and population of the microbial communities in such natural electrogenic environments because the formation of electromotive forces and electroconductive fields is temporally and spatially limited and unstable, and the redox gradient usually fuels chemosynthetic production and populations that would overwhelm the electrosynthesis. Hence, most of the investigations of microbial electrosynthesis are still at the stage of laboratory experiments and are based on several representative species and enrichments.

The deep-sea hydrothermal system is a well-known environment of chemosynthetic ecosystems [16]. Biomass production is derived from the redox disequilibrium between hydrothermal fluid and seawater. Many chemolithoautotrophic microorganisms can use hydrothermal fluid components (such as H_2_S and H_2_) as electron donors and seawater components (such as O_2_ and NO_3_^-^) as electron acceptors. The electron flow from hydrothermal fluid to seawater is catalysed by not only microbial communities, but also minerals, such as sulfide deposits [17]. Sulfide ores containing pyrite and chalcopyrite, which are formed and accumulate around deep-sea hydrothermal vents, efficiently catalyse H_2_S oxidation and O_2_ reduction [17]. As these ores were also found to have long-range electrical conduction [17], the deep-sea hydrothermal field is one of the prominent natural electrogenic environments and acts as a natural electrochemical fuel cell [18, 19]. In this fuel cell system, ores exposed to the hydrothermal fluids act as anode materials and accept electrons from reductants, such as H_2_S (HS^-^). The electrons extracted from the reductants are transported via these conductive ores to distant seawater-rock interfaces, and drive cathodic reactions with seawater [18**–**20]. Therefore, it is hypothesised that entire parts of the outer surfaces of hydrothermal mineral deposits function as cathodes for electrotrophic microbial growth [20, 21], potentially fostering electrosynthetic microbial populations. Since hydrothermal fluid and seawater are continuously supplied with sufficient fluidity on the lower and upper surfaces of hydrothermal mineral deposits, the formation of the electron motive force is stably maintained for a long time. This environment is advantageous as an electrogenic environment compared to other natural environments, such as the redox interfaces of sediment/soil under water/air, in which reductants supply is slower. In addition, the uneven electrical resistance of mineral deposits at each location results in redox potential variation across whole surface area of the deposit, providing favourable habitats for diverse electrosynthetic microbial ecosystems [19]. In this study, we attempted to enrich natural electrosynthetic microbial populations in a deep-sea hydrothermal field using an in situ electrochemical cultivation system. This study leads to the first discovery of a possible electrosynthetic microorganism from a deep-sea hydrothermal vent and the potential genetic and molecular mechanisms and indicates to the occurrence of an electrosynthetic microbial ecosystem driven by the geoelectricity of a deep-sea hydrothermal vent.

## Materials and methods

### Deep-sea research expedition

From the 11th to 27th of January 2015, a research cruise (NT15-02) was conducted by the research vessel (RV) Natsushima (JAMSTEC) and the remotely operated vehicle (ROV) Hyper Dolphin (JAMSTEC) in a deep-sea hydrothermal field at the Iheya North original site (27°47.50′N, 126°53.80′E), Okinawa Trough. An artificial hydrothermal vent, C0014G, was constructed by the deep-sea drilling vessel Chikyu during the Integrated Ocean Drilling Program (IODP) Expedition 331 on the seafloor at a water depth of 1050 m in the Iheya North hydrothermal field [22]. C0014G comprised a guide base on the seafloor and a casing pipe inserted from the centre of the guide base into the subseafloor at 117.8 m below the seafloor (mbsf). A 1.5 m diameter lattice disc was placed on the guide base and hydrothermal fluid was discharged from the open outlet pipe at the centre of the lattice disc. The ROV could land on the lattice disc and be operated beside the artificial hydrothermal vent.

### In situ electrochemical cultivation system

**Fig. 1:**
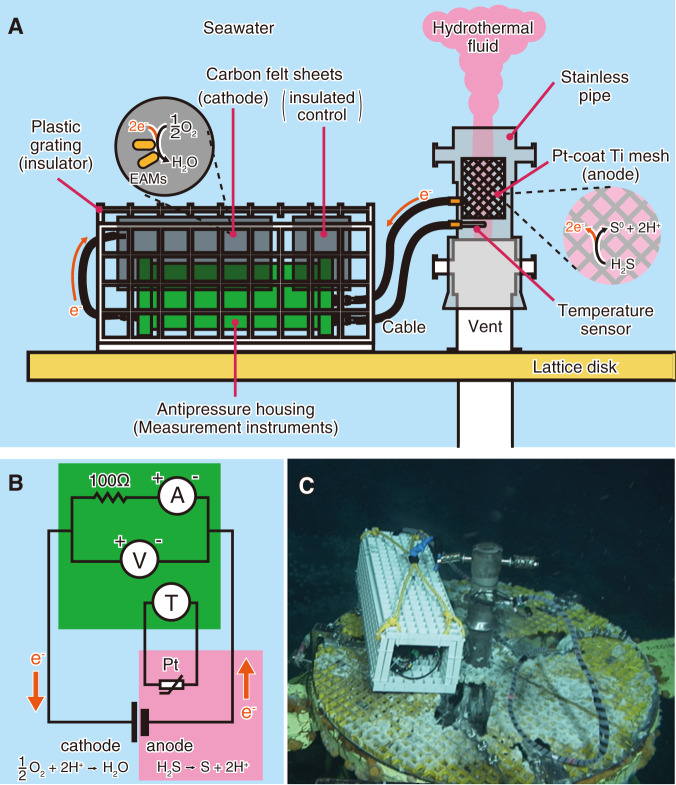
Illustration and photograph of the in situ electrochemical cultivation (ISEC) system. **A** System outline. It was expected that electroactive microorganisms (EAMs) would be enriched on the cathode of the carbon felt sheet, where electrons were supplied from the hydrothermal fluid. **B** Circuit diagram of the system. A ammeter, V voltmeter, T temperature logger, Pt platinum resistor thermometer, green box antipressure housing, cyan box: seawater, magenta box: hydrothermal fluid, orange arrows: electron flow (opposite to current direction). **C** Photograph of the system installed in an artificial deep-sea hydrothermal vent. The water depth was approximately 1050 m.

An in situ electrochemical cultivation (ISEC) system, a fuel cell, was constructed using natural fuels of hydrothermal fluid and seawater as reducing and oxidising agents, respectively (Fig. 1A). A fuel cell system based on the same principle was used in a previous study [18]. A platinum-coated titanium mesh board (100 × 10 cm^2^) was built into a stainless-steel pipe. A titanium wire connected to the mesh board was passed through a small hole on the lateral side of the stainless-steel pipe, which was closed using heat-resistant cement. The titanium wire tip was connected to an underwater cable outside the stainless-steel pipe. A platinum resistor thermometer was also inserted into the stainless-steel pipe through another small hole on the side surface. The stainless-steel pipe, placed at the hydrothermal fluid outlet of the artificial hydrothermal vent, functioned as an anode for the fuel cell system. The underwater cables from the stainless-steel pipe were connected to an antipressure housing, which contained measurement instruments such as a voltmeter with 2.2 MΩ resistance, an ammeter with 10 Ω resistance, a 100 Ω resistor, and a temperature data logger (Fig. 1B). Another underwater cable from the housing was connected to a titanium wire, which was connected to a carbon felt sheet (67 × 60 cm^2^, sample name: EC) as the cathode of the fuel cell system. As a control, another carbon felt sheet (67 × 10 cm^2^, sample name: NC) was placed next to the cathode, but without electrical contact. The carbon felt sheets were sandwiched between plastic gratings to prevent them from contacting other solid conductors. These gratings were placed to cover the antipressure housing.

### Installation of the ISEC system on an artificial hydrothermal vent

The ISEC system was installed on the artificial hydrothermal vent C0014G by using a manipulator on the ROV. The stainless-steel pipe was set on a vent pipe and the hydrothermal fluid passed through the stainless-steel pipe. An antipressure housing covered with plastic gratings was placed on the lattice disc (Fig. 1C). After incubating for 12 days, the ISEC system was collected by the ROV and recovered onto the ship.

### SEM and EDS analyses

For scanning electron microscopy (SEM) observations, approximately 1 cm rock samples were coated with osmium using an osmium plasma coater (POC-3; Meiwafosis; Tokyo, Japan) and observed using a Quanta 450 FEG environmental SEM (FEI; Hillsboro, OR, USA) with 5 kV of acceleration voltage. Energy dispersive spectrometry (EDS) was used to estimate the elemental composition of the rock samples. The SEM–EDS analyses were conducted at 15 keV acceleration voltage.

### XRD analysis

The samples for X-ray diffraction (XRD) analysis were crushed and pulverised using an agate mortar and pestle. Diffraction data were acquired using a MiniFlex II instrument (Rigaku Corporation; Yamanashi, Japan) with a Cu source, 30 kV generator voltage, and 15 mA current. The XRD operating conditions included step scans from 3° to 90° 2θ in 4,350 steps at 2° 2θ/min with a 1.25° divergence slit and 0.3 mm analytical slit. The diffraction data were analysed using the manufacturer’s diffraction evaluation software (PDXL II) in combination with a crystal database from the International Centre for Diffraction Data (https://www.icdd.com/).

### Protein and DNA extraction from carbon felt

The carbon felt sheets (EC and NC) collected from the ISEC system were removed from the plastic gratings on the shipboard (Fig. S3). The felt sheets were separated into three parts as the top and both sides (front and opposite sides of the vent) of the surfaces of the ISEC system. These sheets were cut into several small pieces and stored at −80 °C. The thawed sample (approximately 2 × 2 cm^2^ pieces) were dipped in 0.1 M NaOH (1 mL/cm^2^ sheet), vortexed and heated at 98 °C for 10 min. After centrifugation (10,000 ×g for 5 min at room temperature), the supernatant was collected, and the protein concentration was measured using the bicinchoninic acid method. Six pieces of the carbon felt sheets (2 pieces each from the top and both sides) were used for each analysis of the EC and NC samples. DNA was also separately extracted from the frozen carbon felt samples cut from three places (the top and both sides). Several fibres were selected from a carbon felt piece with tweezers, and DNA was extracted using a DNA extraction kit (PowerSoil DNA Isolation Kit; QIAGEN; Venlo, Netherlands).

### Small subunit rRNA gene-based analysis

Small subunit (SSU) rRNA fragments were amplified using the universal primer set EUB530F-U907R [23], and the amplicons were sequenced using an Ion Torrent PGM sequencer (Life Technologies; Carlsbad, CA, USA) equipped with a 314 chip using 400-base chemistry. For quality filtering, single-end reads were trimmed and filtered using PRINSEQ v0.20.4 [24] with the following parameters: trim_qual_left 20, trim_qual_right 20, and min_len 100. The PCR primer sequences were removed using Cutadapt v1.10 [25]. The resulting sequences were analysed using the QIIME2 v2019.4.0 pipeline [26]. The generated operational taxonomic units (OTUs) were assigned to taxa using the SILVA 138.1 database [27].

### Metagenome analysis

Details of the procedures are described in the supplementary information.

## Results

### Electricity generation with hydrothermal fuel cell on an artificial hydrothermal vent

**Fig. 2:**
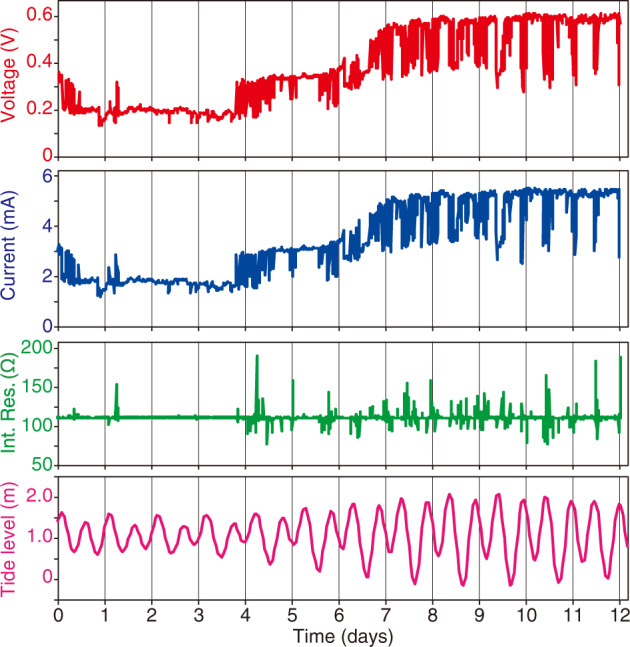
Transitions of the voltage, current and internal resistance of the ISEC system and the tide level in the sea area. The voltage and current were measured by a voltmeter and ammeter connected between the anode and cathode of the ISEC system, respectively. The internal resistance was calculated as the quotient of voltage/current. The tide level was that of the sea surface in the sea area of the hydrothermal field.

We installed an ISEC system on an artificial hydrothermal vent for 12 days (Fig. 1). The anodic electrode, a platinum-coated titanium mesh, was exposed to hot hydrothermal fluid, and the cathodic electrode, a carbon felt sheet, was exposed to cold seawater surrounding the hydrothermal vent. After 12 days, the system was collected onboard, and the collected data were extracted. The temperatures of the hydrothermal fluid and seawater were stable at approximately 314 °C and 4–5 °C, respectively (Table S1). Electricity generation was observed for 12 days (Fig. 2). For the first 2 h after the installation of the system, the fuel cell voltage across the 110 Ω resistor (a 100 Ω resistor and a 10 Ω output impedance in an ammeter) was more than 0.3 V (average: 0.34 V). After 2 hours of incubation, the voltage decreased to approximately 0.21 V and remained constant. The voltage increased after four days of deployment and stabilised at approximately 0.6 V after eight days. The electric current was directly proportional to the voltage change, indicating that the internal resistance was constant with an initial value of 110 Ω in the internal circuit (Fig. 2). The electric current flowed consistently in the direction of electron release from the carbon felt sheet electrode to the seawater. The maximum current density and total charge-transfer density during the 12 days of installation were 13.7 mA/m^2^ and 8.8 × 10^6 ^C/m^2^, respectively. There were often sudden voltage drops during the deployment in a cycle of approximately 12-hour intervals, particularly in the latter half of the 12 days. This cycle corresponds to the semidiurnal high-tide periodicity (Fig. 2).

### Analysis of ore precipitated on the anode

After 12 days of deployment, light and fragile ores were precipitated on the anode surface (Fig. S1A, B). The electric conductivity of the ore sample was not detected (>40 MΩ/cm). XRD analysis indicated that these ores contained anhydrite (CaSO_4_), gypsum (CaSO_4_⋅2H_2_O), and trace amounts of sphalerite (ZnS) and chalcopyrite (CuFeS_2_) (Fig. S2). SEM and EDS indicated that the ore comprised relatively large columnar anhydrite particles and small composite silica and chalcopyrite particles filled the gaps between the anhydrite particles (Fig. S1C, D).

### Microbial community formed on the cathode

**Fig. 3:**
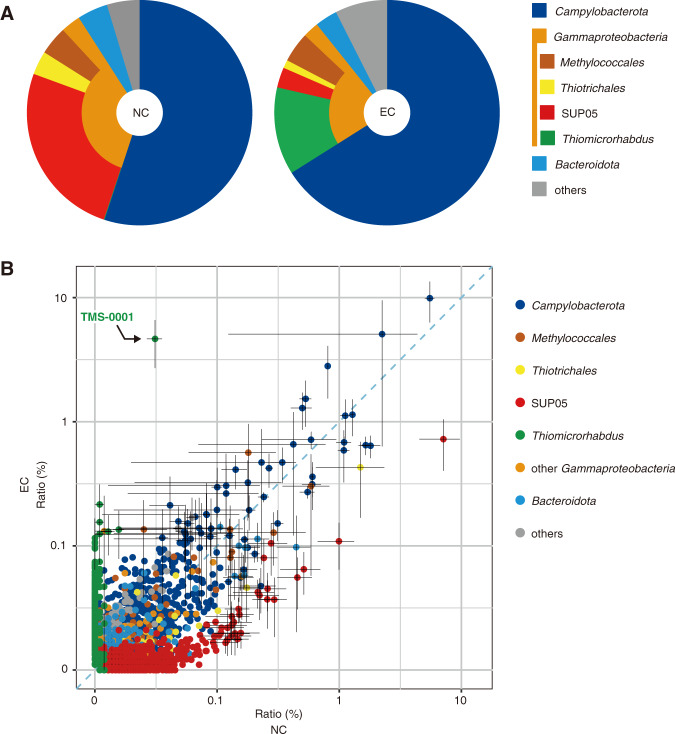
Comparison of the microbial compositions of the ISEC system with and without electricity based on the 16 S rRNA gene. DNA was extracted from the carbon felt sheet of the cathode in the ISEC system (EC) and the insulated sheet as a negative control (NC). Three points on each sheet were analysed. **A** Taxonomic abundance ratios of the EC and NC samples, shown by the average of three replicate analyses. **B** Comparison of the abundance ratios of the operational taxonomic units (OTUs) of the EC and NC samples. Each circle indicates an OTU and is plotted by the average abundance ratio of the three analyses, with the error bars indicating the standard deviation. Vertical axis: EC; horizontal axis: NC.

To examine the effect of the cathodic current on the microbial community, we analysed the microbial populations enriched on the cathode of the ISEC system (samples: EC) (Fig. S3). Another carbon felt sheet, which was set approximately 6–8 cm from the cathode under electrically insulated conditions, was used as a control comprising the nonelectroactive microbial communities adhered to the carbon felt sheet (samples: NC). The DNA assemblages were extracted from three points on each carbon felt sheet (the top and both sides), and the microbial phylotype compositions were determined using SSU rRNA gene amplicon sequencing (Fig. 3A and Table S3). Typical microbial components in the mixing zones of the habitats of deep-sea hydrothermal environments, such as potentially chemoautotrophic H_2_− and sulfur-oxidising bacteria belonging to the *Campylobacterota* and *Gammaproteobacteria* groups, were observed in the DNA assemblages from both carbon felt samples (EC and NC). A comparison of the phylotype compositions between the EC and NC samples indicated different emergent patterns and abundances of *Pseudomonadales* and *Thiomicrospirales* members belonging to *Gammaproteobacteria*. In the NC samples, the *Thioglobaceae* sequences were dominated by the SUP05 group (25.5 ± 12.6% of total abundance) members of the family *Thioglobaceae*. However, in the EC samples, the SUP05 members were less abundant than in the NC sample (2.9 ± 1.9% of total abundance). In contrast, members of the genus *Thiomicrorhabdus*, belonging to the family *Thiomicrospiraceae*, increased the abundance in the EC samples (12.5 ± 7.3% of total abundance), although they were very minor components in the NC samples (<0.1%). In particular, an OTU (TMS-0001) of *Thiomicrorhabdus* dominated the microbial phylotype composition in the EC samples (4.7 ± 3.4% of total abundance) (Fig. 3B). In addition, the protein contents on the carbon felt samples were measured as a proxy for biomass estimation and were found to be 4.5 ± 1.5 g/m^2^ for the EC samples and 3.0 ± 0.9 g/m^2^ for the NC samples (Table S2). This implies that a higher biomass production of the microbial communities on the carbon felt sheet may be promoted by the geoelectricity.

### Genome reconstruction by metagenomic analysis

Metagenomic analysis of the microbial communities on the carbon felt samples (Table S4) resulted in approximately 228,000 contigs with a total size of 297 Mb, of which 77 contigs had the 16 S rRNA gene corresponding to the results of amplicon analysis, including *Campylobacterota*, *Thiomicrorhabdus*, and the SUP05 cluster bacterium (Fig. S4). One of them, with a high sequence coverage of 16 S rRNA genes (1209), was assigned as a member of *Thiomicrorhabdus* and had an identical sequence to that of the OTU of TMS-0001 (Table S5), which was enriched in the EC sample (Fig. 3B). The metagenome-assembled genome (MAG) for this bacterium, ISEC-1, was constructed from metagenomic binning (Table S6). The draft genome comprised 23 contigs with 2,263,286 bp and a 40.9% GC content. The completeness of the genome was estimated to be 99.7%, as determined by the single copy marker genes.

The MAG ISEC-1 was compared with the genomes of nine other *Thiomicrorhabdus* and two closely related *Hydrogenovibrio* strains (Table S7). The MAG ISEC-1 had the smallest genome size (2.26 Mb) and the lowest number of coding sequences (CDSs) (1978) among the *Thiomicrorhabdus* strains. Phylogenetic based on 29 single-copy genes (Table S8 and Fig. S5A) indicated that the MAG ISEC-1 was closely related to *Tmr. arctica*, a psychrophilic, chemolithoautotrophic sulfur-oxidising bacterium isolated from marine Arctic sediments [28]. However, the average nucleotide identity (ANI) of the whole genome sequence and the average amino acid identity (AAI) of all CDSs (Fig. S5B) between them were 69−73% and 67−74%, respectively, which are lower than the levels of identification for the same species (>94% and >95%, respectively) [29, 30]. These results indicate that the MAG ISEC-1 can represent a new species in the genus *Thiomicrorhabdus*.

**Fig. 4:**
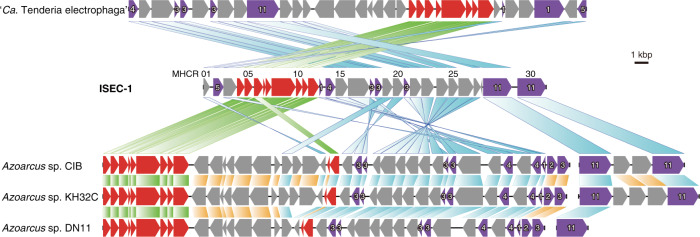
Putative EET gene clusters in the MAG ISEC-1 and other strains. The protein ID (MHCR 01-30) corresponds to the coding sequence (CDS) code of the MHCR gene cluster in the MAG ISEC-1, corresponding to the CDS list shown in Table S9. The colours in the gene box indicate the following; red: cytochrome *c* biogenesis protein, purple: cytochrome *c*, and grey: function unknown protein. The white number indicate the number of hemes included in the cytochrome *c* proteins.

The MAG ISEC-1 showed many genetic features similar to the genomes of other *Thiomicrorhabdus*; however, several gene clusters that had not been found in other *Thiomicrorhabdus* genomes were present in the MAG ISEC-1. In particular, a gene cluster containing many multi-heme cytochrome *c* CDSs was notable (Fig. 4 and Table S9). This cluster comprised 30 genes, eight of which encoded cytochrome *c*, seven of which possessed multiple (3–11) hemes. Subcellular localisation prediction using the PSORTb and CELLO systems suggested that all cytochrome *c* proteins were localised in the periplasm and extracellular space. Most of other proteins in the cluster might be localised in the inner and outer membrane, including the proteins for the cytochrome *c* biogenesis systems I (CcmA-I) [31] and II (CcsXAB) [32]. Other membrane proteins were function-unknown, but one of them had a beta-barrel structure. Two membrane proteins and one periplasmic protein had 6-blade beta-propeller structures. We called the cluster multi-heme cytochrome *c*-rich (MHCR) gene cluster. Exploring this gene cluster in the genomic and metagenomic data, this cluster was not found in the genomes of other *Thiomicrorhabdus* strains but was completely or partially shared with other genomes in certain taxonomic lineages of the phylum *Proteobacteria* (Table S10 and Fig. S6). In particular, several *Gammaproteobacteria*, including ‘*Ca*. Ten. electrophaga’, and several *Betaproteobacteria*, such as *Azoarcus* sp. strains (DN11, KH32C, and CIB), showed high conservation for the gene set possession. Moreover, ‘*Ca*. Ten. electrophaga’ was enriched in the biofilms on a biocathode of an electrochemical reactor operated in the laboratory [11, 33]. It has been reported that ‘*Ca*. Ten. electrophaga’ uses the EEU pathway for electrosynthetic activity [11, 13, 33].

Except for the MHCR gene cluster, the genome of ISEC-1 basically showed similar features to the genomes of other *Thiomicrorhabdus* strains in central carbon and energy metabolism but with some differences. Possession of the gene set for the Calvin–Benson–Bassham (CBB) cycle indicates the strain ISEC-1 exhibits autotrophy similar to other obligately autotrophic *Thiomicrorhabdus* strains [34]. The MAG ISEC-1 indicates the incomplete tricarboxylic acid (TCA) cycle. In other *Thiomicrorhabdus* strains, some NADH-dependent dehydrogenases (isocitrate EC 1.1.1.41, 2-oxoglutarate EC 1.2.4.2/2.3.1.61/1.8.1.4, and malate EC 1.1.3.7) are absent and replaced by alternative enzymes such as NADP^+ ^isocitrate dehydrogenase (EC 1.1.1.42), malate:quinone oxidoreductase (EC 1.1.5.4), and a set of 2-oxoglutarate decarboxylase (EC 4.1.1.71) and succinate semialdehyde dehydrogenase (EC 2.2.1.16), and may catalyse the complete oxidative TCA cycle [34]. The MAG ISEC-1 also possessed NADP^+^ isocitrate dehydrogenase, malate:quinone oxidoreductase, and succinate semialdehyde dehydrogenase, but not 2-oxoglutarate decarboxylase. There is no other bypass pathway between 2-oxoglutarate and succinyl-CoA such as 2-oxoglutarate:ferredoxin oxidoreductase (EC 1.2.7.3) in the MAG ISEC-1. Since malate:quinone oxidoreductase does not catalyse the reduction of oxaloacetate to malate [35], the TCA cycle cannot be used as the oxidative/reductive branched pathway. Although the MAG ISEC-1 possesses genes for the oxidation of succinyl-CoA to oxaloacetate, it is rarely used except when the substrates are supplied by the catabolic degradation of organic compounds [36].

Regarding energy metabolism, MAG ISEC-1 indicates the sulfur-oxidation ability based on the Sox system (SoxXYZABCD), which is the multienzyme system for the oxidation of inorganic sulfur compounds, such as sulfide, elemental sulfur, and thiosulfate [37]. Sulfide:quinone oxidoreductase (Sqr) is also contained in the MAG ISEC-1 but not a dissimilatory sulfite reductase (Dsr) system, similar to other *Thiomicrorhabus* strains. One gene set encoding [NiFe] hydrogenase (EC 1.12.5.1; H_2_-uptake type hydrogenase group 1b) was found in the MAG ISEC-1 [38]. This hydrogenase is not generally found in gammaproteobacteria but is found in *Thiomicrospira microaerophila* and *Hydrogenovibrio* crunogenus XCL-2, although these species cannot use hydrogen as an electron donor and the physical role of hydrogenase is unclear [34]. There is no apparent gene for the use of other inorganic electron donors as the energy source in the MAG ISEC-1. A gene set encoding cbb_3_-type cytochrome *c* oxidase as the sole terminal enzyme of the respiratory chain indicates that strain ISEC-1 adapts microaerobic respiration and does not use any other terminal electron acceptor.

The MAG ISEC-1 also possessed genes (*cysNDCHJIEKM*) for sulfur assimilation from sulfate. Most other *Thiomicrorhabdus* members only have genes (*cysIJKM*) that allow sulfur assimilation from sulfite, sulfide, and thiosulfate [39]. These species depend on the Sox system for not only energy metabolism but also sulfur assimilation [34]. The sulfur assimilation pathway from sulfate has been reported in *Thiomicrorhabdus* sp. Milos-T2 and *Thiomicrorhabdus* sp. GH MAG [40, 41]. This indicates that these species, including strain ISEC-1, can use sulfate as a sulfur source.

## Discussion

In this study, we installed an ISEC system on an artificial deep-sea hydrothermal vent to enrich natural electrotrophic microbial populations. Electricity was generated by the electromotive force between the hydrothermal fluid and seawater. Electrons moved from the hydrothermal fluid to an anodic mesh board, passed through the internal circuit in the system, and were released via a cathodic carbon felt sheet to the seawater. We successfully monitored the power generation over 12 days. The electrode voltage gradually increased and remained constant at approximately 0.6 V, which is a comparable value to the difference in redox potentials (∆0.52 V) between hydrothermal fluids (−0.04 V vs. SHE) and ambient seawater (+0.48 V vs. SHE) measured previously [18]. These results indicated that the system effectively exerted the potential of electromotive force in the deep-sea hydrothermal vent. In this case, the anode and cathode potentials were expected to be similar to the potentials in hydrothermal fluid and seawater, respectively [18, 19]. The gradual increase in cell voltage and current after 4 days of ISEC operation was likely due to the enhancement of cathodic activity by the function of EAMs colonising the cathode, the limiting electrode, as reported for the cell voltage progression with anode community development in microbial fuel cells [42] or as the increased open circuit potential by the cathodic enrichment of ‘*Ca*. Ten. electrophaga’ [43, 44].

Although we found precipitated ores on the anode of the ISEC system after 12 days of deployment, their effect on the power output was unclear. The dominant ore components, such as anhydrite and gypsum, were insulators and only a few conductors, such as chalcopyrite, were observed. There is no evidence that these ores contributed to the power generation; however, no significant inhibition was observed. Most likely, the deposition of insulating ores on the anode does not interfere with the power output of the system if there are some gaps for fluid to pass through the ore because the reaction velocity of hydrogen sulfide oxidation on the anode surface is high enough for oxygen reduction on the cathode [18]. During the process of hydrothermal vent chimney formation, anhydrite and gypsum are first precipitated, which are then replaced by zinc sulfide and lead sulfide, thus enriching chalcopyrite and pyrite [45, 46]. Therefore, as time passes, more chalcopyrite and pyrite might precipitate, which may increase the anode conductivity and surface area.

In this study, a half-day cycle of electricity output was observed corresponding to the tidal cycle (Fig. 2). Tidal stream velocity on the seafloor was synchronised with the semidiurnal tide periodicity, which was the lowest at flood tide and the highest at the inflection point between flood and ebb tide, of the Iheya hydrothermal field (same area as this study) [47]. In fact, drops in electricity output were observed mainly at flood tide. The amount of hydrothermal vent fluid is also changed in response to the tide cycle, but with the phase lags depending on the permeability under the seafloor [48]. Since the electricity generation rate was limited by the supply of dissolved oxygen from seawater to the cathode rather than the hydrogen sulfide from to the hydrothermal fluid to the anode [18], it is more plausible that a half-day cycle of electricity output was controlled by the tidal stream velocity on the seafloor linked to the tide cycle. This periodic change in geoelectricity generation may affect the function of EAMs on the cathode of the ISEC system.

**Fig. 5:**
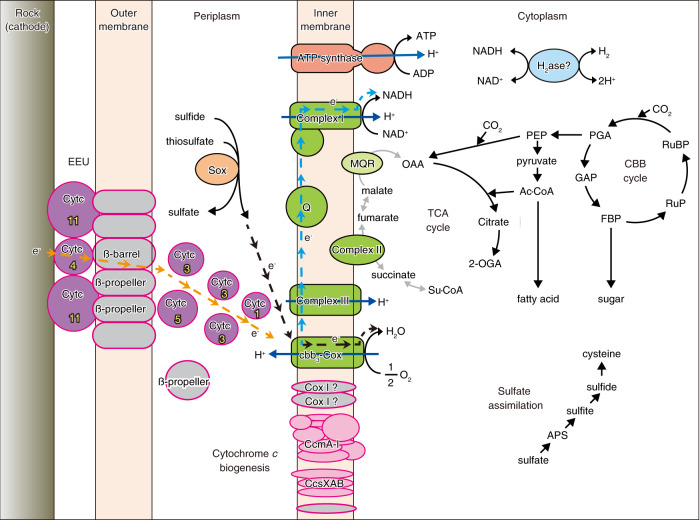
Putative metabolic pathways in the MAG ISEC-1 strain. The dotted arrows indicate the electron flow through the electron transport chain. Orange arrow: EEU pathway. Cyan arrow: Reverse electron transport consuming the proton motive force. The magenta outlined box indicates the protein encoded in the MHCR gene cluster. Purple box: cytochrome *c* including the number of hemes, pink box: cytochrome *c* biogenesis systems, grey box: function-unknown. The positional relationship between each protein is unclear. Abbreviations; Cytc cytochrome c, Sox sulfur oxidation multienzyme system, Q quinone, MQR malate:quinone reductase, Cbb_3_-Cox cbb_3_-type cytochrome *c* oxidase, Cox I cytochrome *c* oxidase subunit I, Ccm cytochrome *c* biogenesis system I, Ccs cytochrome *c* biogenesis system II, H_2_ase hydrogenase, OAA oxaloacetate, 2-OGA 2-oxoglutarate, Su-CoA succinyl-CoA, PEP phosphoenol-pyruvate, Ac-CoA acetyl-CoA, PGA 3-phosphoglycerate, GAP glyceraldehyde 3-phosphate, FBP fructose-1,6-bisphosphate, RuP ribulose-5-phosphate, RuBP ribulose-1,5-bisphosphate, APS adenosine 5’-phosphosulfate.

After 12 days of incubation of the ISEC system, microbial communities seemed to colonise the carbon felt sheets of both the cathode of the fuel cell system and the insulated negative control based on the DNA and protein extraction from the carbon felt samples. A higher protein concentration was obtained from the microbial community on the carbon felt sheets of the ISEC system than that on the control carbon felt sheets, suggesting that the biomass production on the cathode of the ISEC system would be enhanced by the geoelectricity. The overall phylotype compositions of the microbial communities on the carbon felt samples with and without the geoelectricity were dominated by members of *Campylobacterota* and *Gammaproteobacteria* and were similar to the typical compositions often observed in the mixing zones of the habitats of hydrothermal vents [49]. This implies that the chemolithotrophic productivity and population supported by hydrothermal fluid inputs are predominant in the microbial communities, irrespective of the presence or absence of electricity. However, a detailed comparison of the phylotype compositions of the two samples showed that the specific enrichment of the *Thiomicrorhabdus* population within *Thiomicrospiraceae* was induced by the geoelectricity. This group contains autotrophic microaerophilic H_2_− and sulfur-oxidisers and is ubiquitous in the global deep-sea and hydrothermal systems [50, 51]. The genome sequence reconstructed from an enriched *Thiomicrorhabdus* population, MAG ISEC-1, possessed typical central carbon and energy metabolic pathways common to the genomes of other members of the genus *Thiomicrorhabdu*s (Fig. 5). The presence of genes for the CBB cycle, Sox system, Sqr, and cbb_3_-type cytochrome *c* oxidase suggests that strain ISEC-1 is capable of autotrophic growth with sulfur oxidation. Nevertheless, the MAG ISEC-1 possessed the MHCR gene cluster (Fig. 4 and Table S9). Almost all genes in the MHCR gene cluster of ISEC-1 were highly homologous to the genes that have been predicted to be involved in EEU pathway of ‘*Ca*. Ten. electrophaga’, which has been enriched on the biocathode of electrochemical reactors [11]. Other bacteria belonging to *Betaproteobacteria*, *Azoarcus* sp. strain KH32C [52], DN11 [53], and CIB [54] isolated from agricultural fields of paddy rice and soybean, gasoline-contaminated groundwater, and culture medium of another *Azoarcus* strain, respectively, have similar genes to the MHCR gene cluster in the MAG ISEC-1. Although these *Azoarcus* strains have not been identified as EAMs, previous studies have shown that *Azoarcus* members are the predominant components of electroactive microbial communities in several bioelectrochemical enrichments [55, 56]. The comparative genomic analysis indicates that within the genus *Thiomicrorhabdus* this MHCR gene cluster is only found in the MAG ISEC-1. However, it is unclear whether this is due to horizontal gene transfer (HGT) acquisition or an absence in phylogenetically related genomes. One of the most extensively studied EET pathways, the MtrCAB system, is also sporadically distributed across a wide range of microbial components and environments, and the high mobility of this gene set suggests the HGT-mediated evolution scenario [57]. Similar to the MtrCAB system, possession of the MHCR gene cluster may be used as an indicator of EEU ability exploration (Table S10).

Since strain ISEC-1 was specifically enriched on the cathodic electrode, we hypothesise that the bacterium can electrotrophically grow with the electrons released from the cathode as an electron source via the EET pathway and can fix CO_2_ as a carbon source, namely, is capable of electrosynthesis. The gene repertoire of ISEC-1 (e.g., Sox system) points to the capability of chemolithoautotrophic growth to reduce the inorganic sulfur compounds, such as H_2_S, from hydrothermal fluids. However, the specific enrichment of MAG ISEC-1 bacterium with geoelectricity suggests that electrotrophic metabolism may effectively contribute to the growth of MAG ISEC-1 bacterium on the carbon felt sheets. The MAG ISEC-1 also possesses a [NiFe] hydrogenase gene. Reversible hydrogenase uses NADH as the redox partner in the cytoplasm [58] and maintains redox balance in highly variable environments [34]. Strain ISEC-1 was enriched with the electromotive force generated by the potential difference between the hydrothermal fluid and ambient seawater, of which the maximum reducing power depends on the redox potential of the hydrothermal fluid and is estimated to be between −0.04 and −0.10 V (vs. standard hydrogen electrode) [18, 19]. This potential is not enough to produce H_2_ on the cathode. Thus, the cathodic H_2_ production of our ISEC system and the hydrogenotrophic growth of MAG ISEC-1 bacterium directly utilising the cathodic H_2_ with the function-unknown hydrogenase seem to be unlikely, although it cannot be excluded that strain ISEC-1 can chemolithtrophically grow with H_2_ as well as the reductive sulfur compounds provided from the hydrothermal fluids.

Since the redox potential provided from hydrothermal fluid is also not sufficient to reduce NAD^+^, strain ISEC-1 is considered to use reverse electron transport by consuming proton motive force, which is achieved through O_2_ respiration. A similar energy metabolism mechanism of the EEU pathway with reverse electron transport has been proposed for the autotrophic iron-oxidiser *A. ferrooxidans* [10], which consumed 198.1 mol electrons from Fe^2+^ to fix 1 mol CO_2_ [59]. In our ISEC system, the total charge-transfer density during cultivation was 8.8 × 10^6 ^C/m^2^, which was equivalent to 91.2 mol electrons flowing per square metre of cathode. According to the energy conversion efficiency for carbon fixation by *A. ferrooxidans*, it is estimated that 0.46 mol CO_2_ could be fixed by the function of MAG ISEC-1 bacterium on a 1 m^2^ cathode when all the flowing electrons are used for energy metabolism. We found that 4.5 ± 1.5 g/m^2^ protein was measured from the microbial community on the cathode after the ISEC operation. In marine bacteria, it has been reported that the cellular carbon content is equivalent to 86% of the total protein content [60]. Based on these values, the measured protein content of the cathodic microbial community is estimated to correspond to 3.9 g (0.33 mol) of the microbially fixed carbon content per 1 m^2^ cathode. Thus, a considerable amount of carbon fixation may be derived from electrosynthetic populations, such as the MAG ISEC-1 bacterium, using geoelectricity.

Based on the gene repertoire in the genome, strain ISEC-1 possesses an incomplete TCA cycle [34] lacking the step connecting 2-oxoglutarate to succinyl-CoA, and has malate:quinone oxidoreductase (MQO) instead of malate dehydrogenase, which means that oxaloacetate is not reduced to malate [35]. This implies that half of the TCA cycle from succinyl-CoA to oxaloacetate, including complex II, rarely functions in strain ISEC-1. In addition, genome analysis indicated that the ISEC-1 strain can use sulfate as a sulfur source for cysteine synthesis. Most *Thiomicrorhabdus* members use reduced sulfur compounds, such as H_2_S, both for their energy and sulfur (cysteine biosynthesis) sources via the Sox pathway [41]. This feature may be associated with the capability of electrosynthetic growth of the MAG ISEC-1 bacterium in highly H_2_S-depleted but electrogenic habitats, e.g., on the surface area of sulfide deposits far away from hydrothermal fluid discharges, where strain ISEC-1 uses the geoelectricity as the energy source and seawater sulfate as the sulfur source without the function of the Sox system.

In this study, we present the successful enrichment of a potentially in situ electrotrophic *Thiomicrorhabdus* bacterium (as MAG ISEC-1) in an artificial electrochemical cultivation system in a deep-sea hydrothermal system with genomic features that indicate the possibility of electrosynthesis. Since geoelectricity is available in the whole surface areas of natural conductive sulfide deposits in the vicinity of hydrothermal systems under the same principle as fuel cells [19], the temporal and spatial scale of habitats for electrosynthetic microbial populations may be much greater than those of habitats for chemosynthetic populations. In fact, around a deep-sea hydrothermal field, several geobatteries were detected without active hydrothermal vents [61]. In addition, as observed in the microbial community on the cathode of our artificial electrochemical cultivation system, electrotrophs are known to be able to outcompete other chemotrophs under sufficient electricity supply conditions. Although the chemosynthetic microbial populations would mask coexisting electrosynthetic populations in the natural electrogenic environments such as hydrothermal fields, further microbiological exploration in natural electrogenic environments will lead to multiple lines of evidence for electrosynthetic populations and functions behind the chemosynthesis-dominating microbial communities and the discovery of hidden electrosynthetic microbial ecosystems.

We propose that the new species be referred to as ‘*Candidatus* Thiomicrorhabdus electrophagus sp. nov. (e.lec.tro.pha’gus. Gr. n. *elektron*, amber, used in science for a negative charge unit; Gr. masc. n. *phagos*, an eater; N.L. masc. n. *electrophagus*, an electron eater), which was enriched by an ISEC system installed at an artificial deep-sea hydrothermal vent. Its genome suggested that the bacterium possessed EEU ability. In the future, it will be necessary to verify whether the bacterium can proliferate using electric energy obtained through the EEU pathway.

## Supplementary information

Supplementary Information

Table S9

Table S10

## Data Availability

The 16 S rRNA gene amplicon sequence data and metagenomic data from the ISECs and the enriched biofilms are available in the DNA Data Bank of Japan (DDBJ) Sequenced Read Archive under accession numbers DRA013670 and DRA013696, respectively. The genome sequences of strain ISEC-1 were deposited in DDBJ/EMBL/GenBank under the following accession numbers: BQXR01000001- BQXR01000023. These data can be found under bioproject number PRJDB13230.
