# Supplementary material for: In situ electrosynthetic bacterial growth using electricity generated by a deep-sea hydrothermal vent
Source: ISME J. 2022 Sep 23;17(1):12–20. doi: 10.1038/s41396-022-01316-6 (PMC9751133; doi:10.1038/s41396-022-01316-6)
Supplement: Supplementary file 1 — Supplementary Information [file 41396_2022_1316_MOESM1_ESM.pdf]

Supplementary Information for the research article:

In situ electrosynthetic bacterial growth using electricity generated by a deep-sea hydrothermal vent

Masahiro Yamamoto,[1*] Yoshihiro Takaki,[1] Hiroyuki Kashima,[1] Miwako Tsuda,[1] Akiko Tanizaki,[1] Ryuhei Nakamura[2,3] and Ken Takai[1]

[1]Institute for Extra-cutting-edge Science and Technology Avant-garde Research (X-star), Japan Agency for Marine-Earth Science and Technology (JAMSTEC), Yokosuka, Japan.

[2]Center for Sustainable Resource Science, RIKEN, Wako, Japan.

[3]Earth-Life Science Institute (ELSI), Tokyo Institute of Technology, Tokyo, Japan.

Table S1. Temperature of the hydrothermal fluid and ambient seawater.

	Temperature (˚C)	Number of measuring times	Measuring period	Measuring interval
Hydrothermal fluid	313.7 ± 0.1	107	54 hours	30 min
Ambient seawater	4.1 ± 0.0	73	36 min	30 sec
	4.6 ± 0.2	23	11 min	

Table S2. Protein concentrations on the carbon felt sheet surfaces of the ISEC system.

Sample location*[1]	Protein concentration (g/m^2)	
	NC	EC
Top	4.39	6.04
	3.48	6.88
Side in front of the vent	3.11	3.39
	2.99	4.17
Side opposite the vent	2.28	3.62
	1.46	2.87
Average	2.95±0.92	4.50±1.50

*[1] Represents where the pieces of the carbon felt sheet were cut out from the ISEC system surface.

Table S3. Numbers and ratios of the OTUs in the microbial composition of the in ISEC system.

Taxon	NC				EC			
	1*[1]	2	3	average±s.d.	1	2	3	average±s.d.
Campylobacterota	25308 (68.3%)	15537 (43.5%)	19850 (53.3%)	20232±3998 (55.1±10.2%)	18719 (65.3%)	21812 (71.2%)	20245 (61.6%)	20259±1263 (66.0±4.0%)
Proteobacteria								
Gammaproteobacteria								
Methylococcales	2866 (7.7%)	807 (2.3%)	904 (2.3%)	1526±949 (4.1±2.5%)	2130 (7.4%)	1408 (4.6%)	363 (1.1%)	1300±725 (4.4±2.6%)
Thiotrichales	631 (1.7%)	759 (2.1%)	2342 (6.3%)	1244±778 (3.4±2.1%)	232 (0.8%)	670 (2.2%)	136 (0.4%)	346±232 (1.1±0.8%)
Pseudomonadales								
Thioglobaceae								
SUP05	3942 (10.6%)	14830 (41.5%)	9030 (24.3%)	9267±4448 (25.5±12.6%)	115 (0.4%)	1019 (3.3%)	1657 (0.5%)	930±633 (2.9±1.9%)
other *Thioglobaceae*	43 (0.1%)	183 (0.5%)	134 (0.4%)	120±58 (0.3±0.2%)	9 (0.0%)	22 (0.1%)	45 (0.1%)	25±15 (0.1±0.0%)
other *Pseudomonadales*	67 (0.2%)	46 (0.1%)	45 (0.1%)	53±10 (0.1±0.0%)	125 (0.4%)	74 (0.2%)	58 (0.2%)	86±29 (0.3±0.1%)
Thiomicrospirales								
Thiomicrospiraceae								
Thiomicrorhabdus	22 (0.1%)	25 (0.1%)	22 (0.1%)	23±1 (0.1±0.0%)	3776 (13.2%)	1026 (3.4%)	6940 (21.1%)	3914±2416 (12.5±7.3%)
other *Thiomicrospiraceae*	21 (0.1%)	7 (0.0%)	3 (0.0%)	10±8 (0.0±0.0%)	6 (0.0%)	12 (0.0%)	16 (0.0%)	11±4 (0.0±0.0%)
other *Gammaproteobacteria*	866 (2.3%)	654 (1.8%)	1028 (2.8%)	849±153 (2.3±0.4%)	614 (2.1%)	805 (2.6%)	259 (0.8%)	559±226 (1.9±0.8%)
Bacteroidota	1495 (4.0%)	1261 (3.5%)	2097 (5.6%)	1618±352 (4.4±0.9%)	803 (2.8%)	1408 (4.6%)	764 (2.3%)	992±295 (3.2±1.0%)
others	1779 (4.8%)	1609 (4.5%)	1758 (4.7%)	1715±76 (4.7±0.1%)	2150 (7.5%)	2358 (7.7%)	2368 (7.2%)	2292±100 (7.5±0.2%)
Total	37040 (100%)	35718 (100%)	37213 (100%)	36657±668 (100%)	28679 (100%)	30614 (100%)	32851 (100%)	30715±1705 (100%)

*[1] Sample number indicates the position of the carbon felt sheet at the ISEC system; 1: top, 2: side in fron of the vent, 3: side opposite the vent.

Table S4. Assembly statistics of the ISEC metagenome.

Contents	ISEC metagenome
Total size of sequence reads (EC and NC samples)	9.05 Gb (4.38 Gb + 4.67 Gb)
Number of contigs (> 500 bp)	228,342
Total length	296.6 Mb
N50	1302 bp

Table S5. Top 10 contigs with a high coverage of 16S rRNA genes in the ISEC metagenome.

Contig	Length of contig (bp)	Average coverage of 16S rDNA	Top hit of organism
ctg000266	2665	2284	*Campylobacterota: Sulfurimonas*
ctg002120	1233	1229	*Campylobacterota Sulfurovum*
**ctg000168**	**5988**	**1209**	***Gammaproteobacteria: Thiomicrorhabdus**** **[1]**
ctg000169	5673	947	*Gammaproteobacteria:* SUP05 cluster
ctg000269	715	635	*Campylobacterota: Sulfurovum*
ctg000268	1168	235	*Campylobacterota: Sulfurovum*
ctg002064	2553	188	*Gammaproteobacteria: Methylococcales*
ctg000970	2720	134	*Gammaproteobacteria: Thiotrichaceae*
ctg011804	522	108	*Campylobacterota: Sulfurimonas*
ctg010246	2300	102	*Campylobacterota: Sulfurimonas*

*[1] Contained 16S rRNA gene sequence that showed 100% identity to OTU TMS-0001 .

Table S6. Summary statistics of the MAG ISEC-1.

Contents	MAG ISEC-1
Number of contigs	23
Total length	2,263,286 bp
N50	229,053 bp
GC content	40.9 %
Average coverage	274
Relative abundance*[1]	7.26 %
**Number of genes**	
CDS	1,978
Copy number of the rRNA operon*[1]	4
tRNA	47
Other RNA	2
**Evaluation*[1]**	
Completeness*[2]	99.7 %
Contamination*[2]	0.3 %

*[1] Estimated from the proportion of reads mapped to the genome.
*[2] The genus *Thiomicrorhabdus* belongs to the family *Piscirickettsiaceae* in the NCBI taxonomy database. The evaluation of MAG ISEC-1was determined based on the lineage-specific marker sets of the family *Piscirickettsiaceae* determined with CheckM.

Table S7. Species and related strains used in genome comparison.

Symbol	Organism	#Contigs/ Scaffolds	Size (Mb)	GC (%)	#CDS	Accession*[1]
ISEC-1		23	2.26	40.9	1,978	This study
TmrKp2	*Thiomicrorhabdus* sp. Kp2	1	2.73	39.9	2,449	GCF_000478585.1
TmrArc	*Tmr. arctica* DSM 13458	6	2.55	40.9	1,978	GCF_000381085.1
TmrChi	*Tmr. chilensis* DSM 12352	2	2.44	48.1	2,206	GCF_000483485.1
TmrMil	*Tmr.* sp. Milos-T2	2	2.66	38.2	2,294	GCF_000702325.1
TmrAqu	*Tmr. aquaedulcis* HaS4	2	2.54	45.3	2,181	GCF_004001325.1
TmrInd	*Tmr. indica* 13-15A	1	2.83	41.6	2,421	GCF_004293625.1
TmrSed	*Tmr. sediminis* G1	1	2.37	45.2	2,094	GCF_005885815.1
TmrXia	*Tmr. xiamenensis* G2	1	2.59	48.4	2,318	GCF_013282625.1
TmrHH3	*Tmr.* sp. HH3	29	2.49	52.4	2,316	GCF_013391695.1
HyvMar	*Hydrogenovibrio marinus*	1	2.49	44.1	2,322	GCA_013340845.1
HyvCru	*H. crunogenus*	1	2.45	42.6	2,293	GCA_004786015.1

*[1] NCBI assembly accession.

Table S8. Genes used for the genome tree analysis.

	KO number	gene	Product	Locus tag
1	K00927	*pgk*	phosphoglycerate kinase	ISEC1_P0982
2	K01937	*pyrG*	CTP synthase	ISEC1_P1319
3	K02316	*dnaG*	DNA primase	ISEC1_P1545
4	K02357	*tsf*	elongation factor Ts	ISEC1_P1343
5	K02600	*nusA*	transcription termination/antitermination protein NusA	ISEC1_P1381
6	K02838	*frr*	ribosome recycling factor	ISEC1_P1341
7	K02863	*rplA*	large subunit ribosomal protein L1	ISEC1_P0953
8	K02867	*rplK*	large subunit ribosomal protein L11	ISEC1_P0954
9	K02871	*rplM*	large subunit ribosomal protein L13	ISEC1_P1519
10	K02874	*rplN*	large subunit ribosomal protein L14	ISEC1_P0933
11	K02878	*rplP*	large subunit ribosomal protein L16	ISEC1_P0936
12	K02884	*rplS*	large subunit ribosomal protein L19	ISEC1_P1439
13	K02886	*rplB*	large subunit ribosomal protein L2	ISEC1_P0940
14	K02887	*rplT*	large subunit ribosomal protein L20	ISEC1_P0715
15	K02899	*rpmA*	large subunit ribosomal protein L27	ISEC1_P0907
16	K02906	*rplC*	large subunit ribosomal protein L3	ISEC1_P0943
17	K02931	*rplE*	large subunit ribosomal protein L5	ISEC1_P0931
18	K02933	*rplF*	large subunit ribosomal protein L6	ISEC1_P0928
19	K02935	*rplL*	large subunit ribosomal protein L7/L12	ISEC1_P0951
20	K02946	*rpsJ*	small subunit ribosomal protein S10	ISEC1_P0944
21	K02948	*rpsK*	small subunit ribosomal protein S11	ISEC1_P0921
22	K02952	*rpsM*	small subunit ribosomal protein S13	ISEC1_P0922
23	K02965	*rpsS*	small subunit ribosomal protein S19	ISEC1_P0939
24	K02967	*rpsB*	small subunit ribosomal protein S2	ISEC1_P1344
25	K02982	*rpsC*	subunit ribosomal protein S3	ISEC1_P0937
26	K02988	*rpsE*	small subunit ribosomal protein S5	ISEC1_P0926
27	K02996	*rpsI*	small subunit ribosomal protein S9	ISEC1_P1518
28	K03043	*rpoB*	DNA-directed RNA polymerase subunit beta	ISEC1_P0950
29	K03664	*smpB*	SsrA-binding protein	ISEC1_P0179

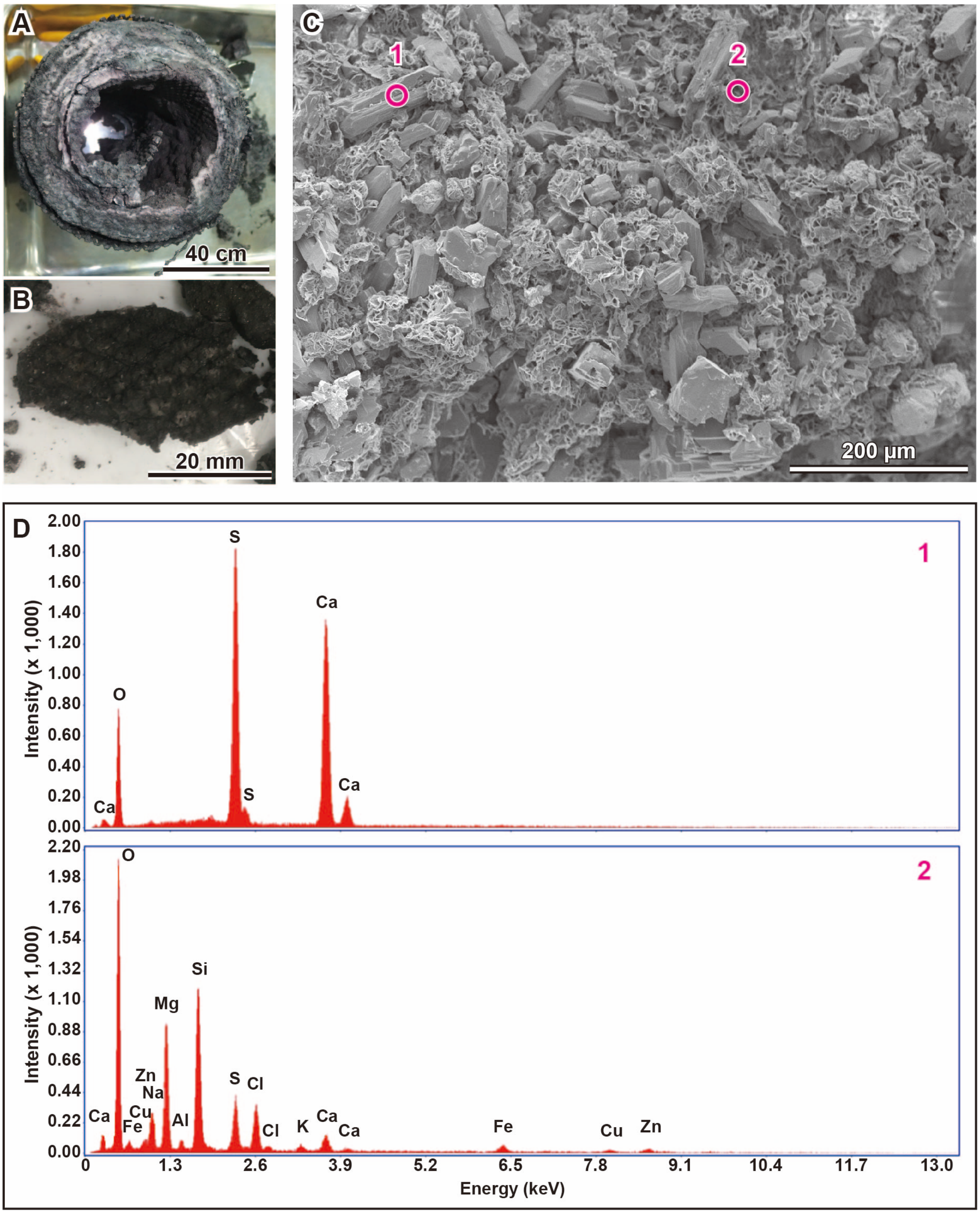

Figure S1

Ore precipitated on the ISEC system anode. Platinum-coated titanium mesh as the anode was exposed to deep-sea hydrothermal fluid for 12 days. (A) Ore filling in the gaps of the anode material. (B) An ore piece. (C) Scanning electron microscopy (SEM) image of the ore surface. Circles indicate the spots of energy dispersive spectrometry (EDS) analysis. (D) EDS analysis of spots on the ore.

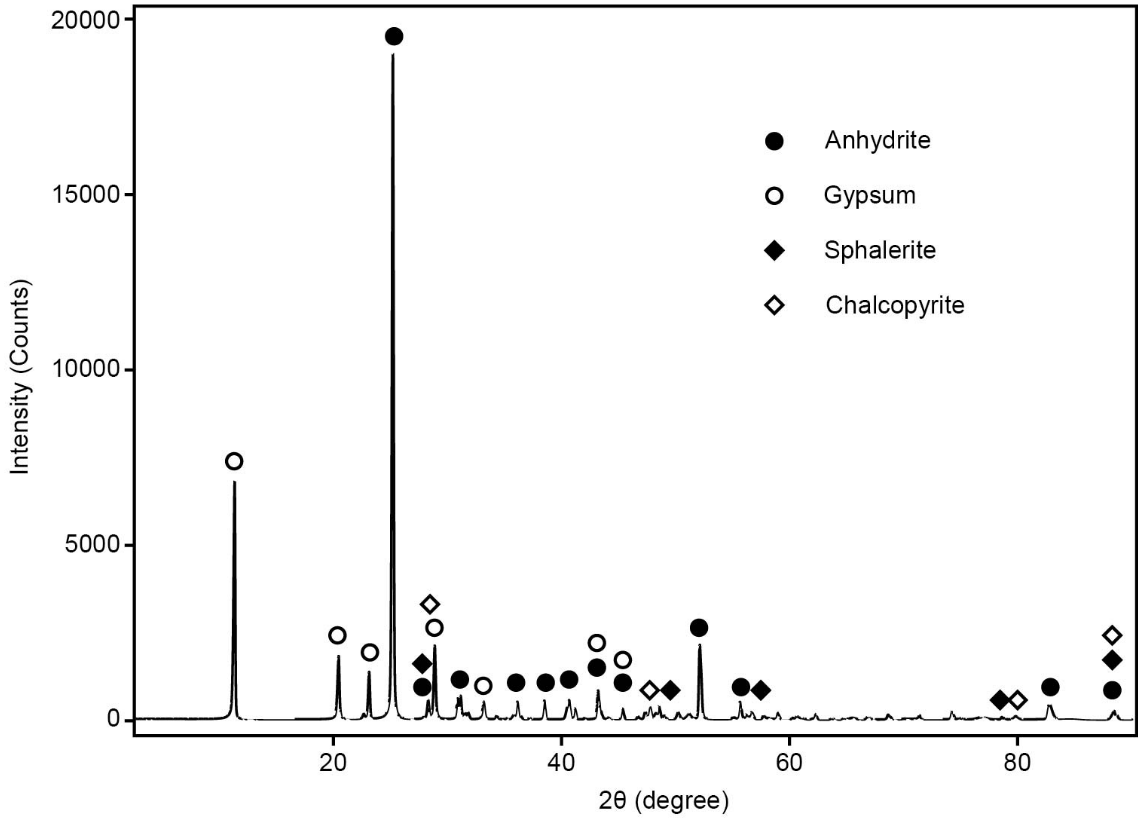

Figure S2
X-ray diffraction (XRD) analysis of the ore on the anode.

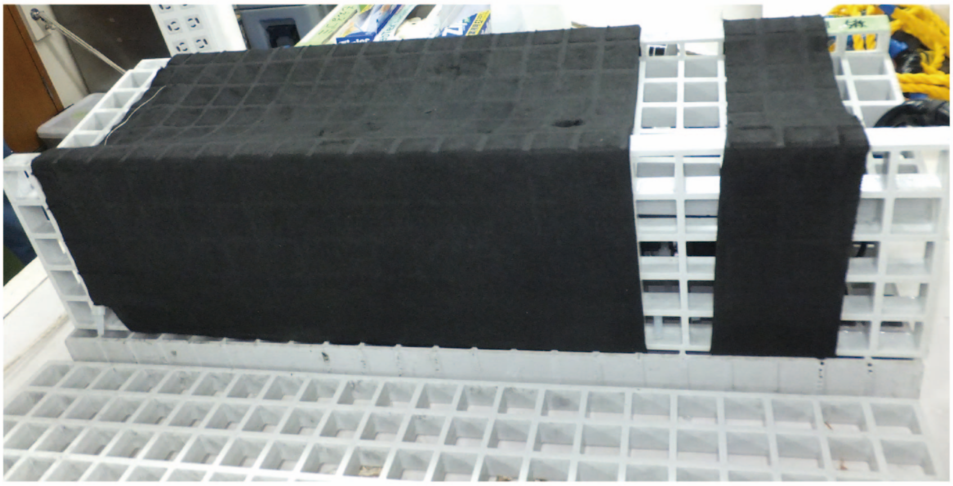

Figure S3
Carbon felt sheets of the ISEC system after cultivation for 12 days. The left sheet is the cathode of the fuel cell, and the right sheet is the insulated negative control. The grid length in the plastic gratings under the carbon felt sheets was 40 mm.

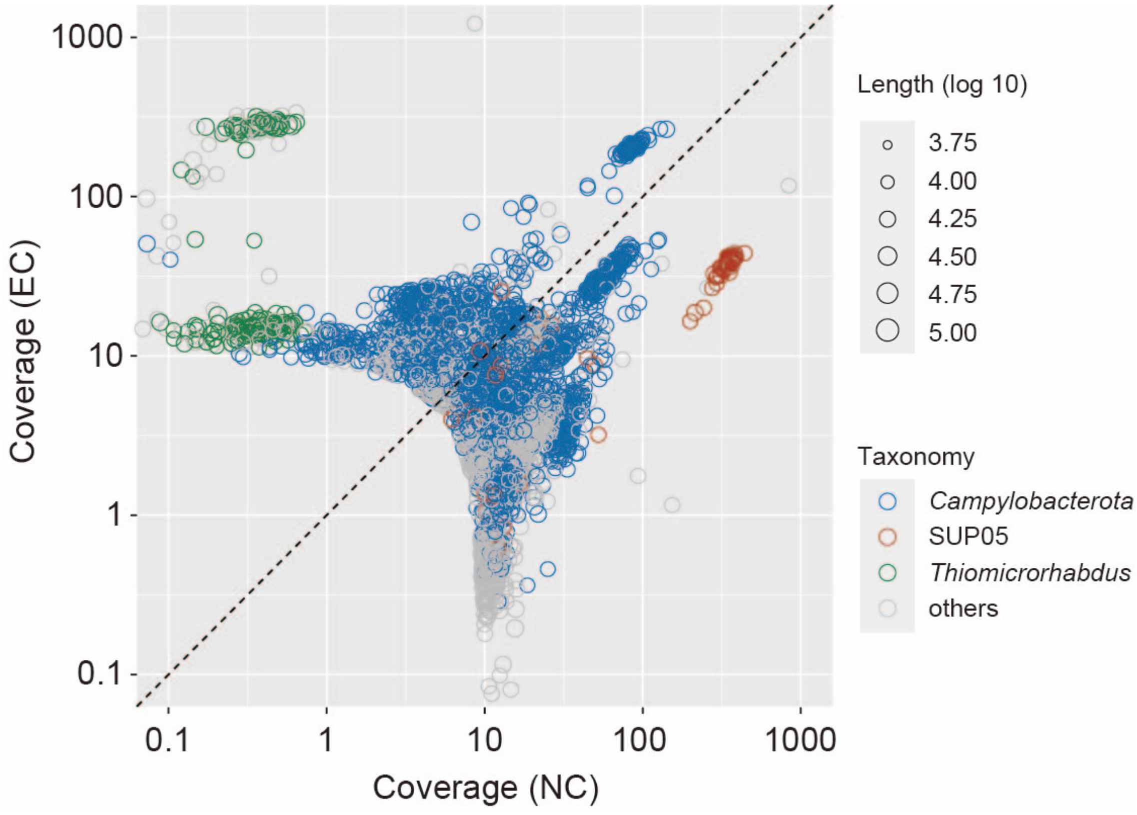

Figure S4

Sequencing coverage of the contigs with each of the EC and NC metagenomes. Circle size indicates contig length. Contigs with low coverage (< 10) were removed from the plot. Circles were coloured by the taxonomy of the best hit organism in Blastx analysis.

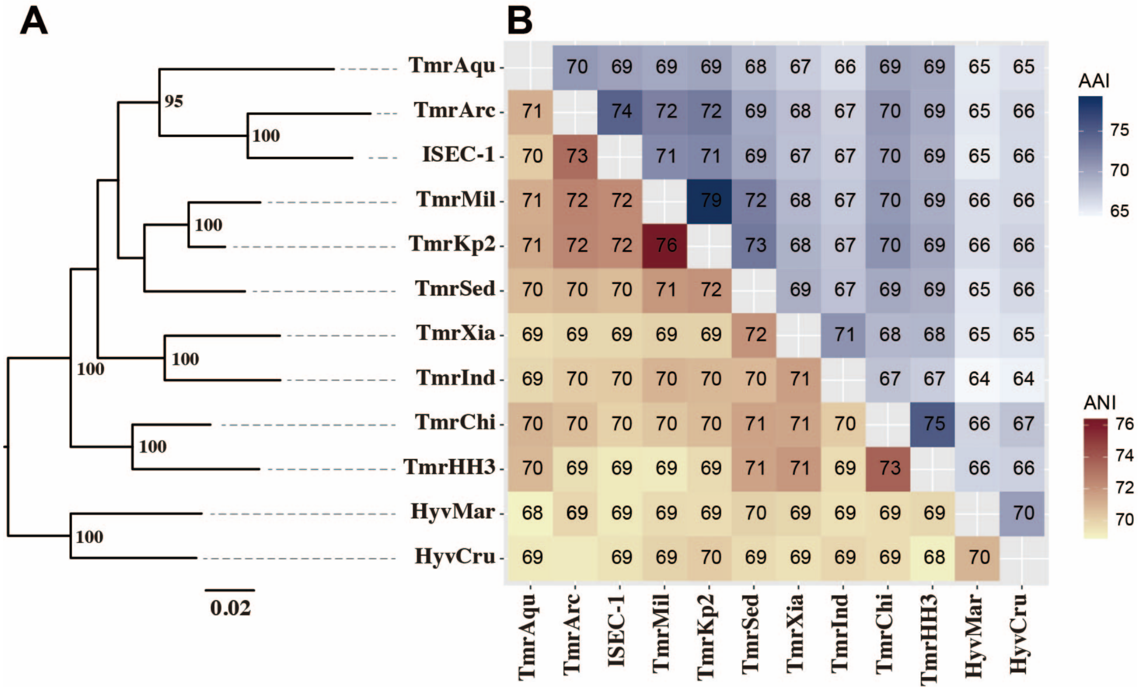

Figure S5

(A) Genome tree based on a concatemer of 29 single copy genes and (B) the ANI/AAI matrix between the genomes of *Thiomicrorhabdus* and related strains. ANI: average nucleotide identity of the whole genome sequence, AAI: average amino acid identity of all coding sequences (CDSs). The genomes used in this analysis are listed in Table S7.

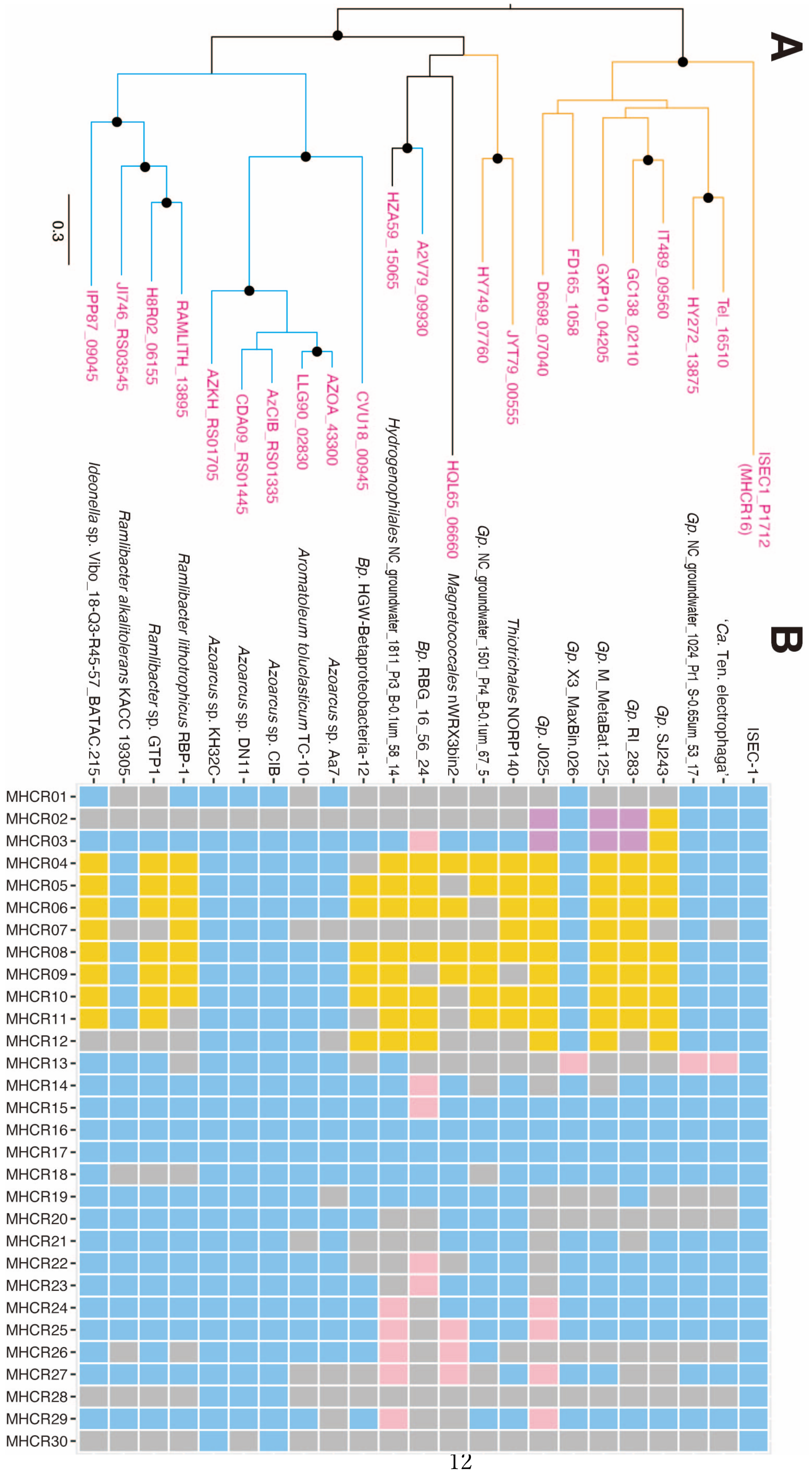

A
B
0.3
ISEC1_P1712 (MHCR16)
Tel_16510
HY272_13875
IT489_09560
GC138_02110
GXP10_04205
FD165_1058
D6698_07040
JYT79_00555
HY749_07760
HQL65_06660
A2V79_09930
HZA59_15065
CVU18_00945
AZOA_43300
LLG90_02830
AzCIB_RS01335
CDA09_RS01445
AZKH_RS01705
RAMLITH_13895
H8R02_06155
JI746_RS03545
IPP87_09045
ISEC-1
'Ca. Ten. electrophaga'
Gp. NC_groundwater_1024_Pr1_S-0.65um_53_17
Gp. SJ243
Gp. RI_283
Gp. M_MetaBat.125
Gp. X3_MaxBin.026
Gp. J025
Thiotrichales NORP140
Gp. NC_groundwater_1501_Pr4_B-0.1um_67_5
Magnetococcales nWRX3bin2
Bp. RBG_16_56_24
Hydrogenophilales NC_groundwater_1811_Pr3_B-0.1um_58_14
Bp. HGW-Betaproteobacteria-12
Azoarcus sp. Aa7
Aromatoleum toluclasticum TC-10
Azoarcus sp. CIB
Azoarcus sp. DN11
Azoarcus sp. KH32C
Ramlibacter lithotrophicus RBP-1
Ramlibacter sp. GTP1
Ramlibacter alkalitolerans KACC 19305
Ideonella sp. Vibo_18-Q3-R45-57_BATAC.215
MHCR01
MHCR02
MHCR03
MHCR04
MHCR05
MHCR06
MHCR07
MHCR08
MHCR09
MHCR10
MHCR11
MHCR12
MHCR13
MHCR14
MHCR15
MHCR16
MHCR17
MHCR18
MHCR19
MHCR20
MHCR21
MHCR22
MHCR23
MHCR24
MHCR25
MHCR26
MHCR27
MHCR28
MHCR29
MHCR30

Figure S6

(A) Phylogenetic tree of the MHCR16 protein from the MAG ISEC-1 and homologous proteins encoded in other bacterial genomes. The magenta characters indicate the CDS locus tags corresponding to Table S10. The orange and cyan lines indicate the lineages of *Gammaproteobacteria* and *Betaproteobacteria*, respectively. The black dot indicates the branch where the bootstrap value is higher than 90. (B) List of microorganisms possessing the same gene set as the MHCR gene cluster. BLASTp with an e-value cutoff of 10^{-5} was used to identify the homologs to the MHCR genes. Microorganism order corresponds to the host of the protein shown in the phylogenetic tree on the left side. The grey box indicates nonpossession of the corresponding gene. The other colour (cyan, yellow, pink, and purple) boxes indicate the possession of the gene, and the same colour box indicates that the genes were located in the same contig. Gp. and Bp. represent *Gammaproteobacteria bacterium* and *Betaproteobacteria bacterium*, respectively.

Materials and Methods

ISEC-1 genome reconstruction from metagenome analysis

Each DNA extracted from the samples with and without the geoelectricity (EC and NC) was applied to metagenome libraries with KAPA Hyper Prep Kit Illumina platforms (Woburn, MA, USA) according to the manufacturer's protocol. Paired-end sequencing was performed using the Illumina MiSeq platform (San Diego, CA, USA) with a 2×300 bp read length. Raw Illumina reads were sequentially processed by using Trimmomatic ver. 0.39 [1] to trim the adaptor sequences and low-quality sequences and Bowtie 2 ver. 2.3.5.1 [2] to remove the PhiX sequences used as internal controls. Two datasets from these libraries were combined and assembled using CLC Genomic Workbench ver. 11 (Qiagen; Hilden, Germany) with the following parameter settings: 64 bp word size, 500 bp bubble size, map read back to the contigs with 0.9 length fraction and a 0.9 similarity fraction.

The ISEC-1 genome was binned and reassembled using the pipeline coded R SCRIPTS (https://github.com/MadsAlbertsen/multi-metagenome) presented in a previous report [3]. Briefly, binning was constructed based on differences in the contigs with respect to GC content, tetranucleotide frequency, and sequencing coverage in the two datasets. The contig coverage was calculated using BBMap (BBMap, Bushnell B., sourceforge.net/projects/bbmap/). Bins of interest were further used as references for the mapping of the cleaned reads. The reads that mapped to the reference were then assembled *de novo* using SPAdes ver. 3.7.1 [4]. Generated contigs were inspected and manually corrected.

Completeness and contamination of the draft genome were calculated with CheckM [5] using a set of lineage-specific genes of *Gammaproteobacteria*. The average nucleotide identity (ANI) and average amino acid identity (AAI) of the sequenced relatives of the genus *Thiomicrorhabdus* were estimated using OrthoANI ver. 1.8.0_121 [6] and CompareM ver. 0.1.2 (https://github.com/dparks1134/CompareM), respectively. To build a genome tree, the amino acid sequences of 29 single-copy marker genes were extracted from the proteomes of the genomes of the ISEC-1 strain, nine *Thiomicrorhabdus* species, and two *Hydrogenovibrio* species. Multiple sequence alignment was performed using MAFFT v7.312 [7], and ambiguously aligned positions were removed using trimAl v1.2 [8]. After concatenating the alignment, a maximum likelihood phylogenomic tree was inferred using RAxML v8.2.9 [9] with the LG4X+G model and 100 bootstrap replications. A best-fit amino acid substitution model was chosen using AMINOSAN v1.0 [10].

Functional annotation

The coding sequences were identified using a combination of MetaGeneMark [11] and Glimmer-MG [12]. The deduced amino acid sequences were subjected to a BLASTP search against the NCBI nr protein database, and their functional annotations were manually assigned based on the Kyoto Encyclopedia of Genes and Genomes (KEGG) Orthology (KO) database. tRNAscan-SE v1.3.1 [13] was used to identify tRNA genes, whereas rRNA and other noncoding RNAs were identified by searching the corresponding Rfam profiles using Infernal ver. 1.1.4 [14]. Functional transmembrane domains and signal peptide sequences in proteins were predicted using TMHMM ver. 2.0 [15] and SignalP ver. 5.0b [16], respectively. Protein subcellular localisations were predicted using PSORTb ver. 3.0 [17] and CELLO ver. 2.5 [18].

References

1. Bolger AM, Lohse M, Bioinformatics BU. Trimmomatic: a flexible trimmer for Illumina sequence data. *Bioinformatics* 2014; **30**: 2114–2120.
2. Langmead B, Salzberg SL. Fast gapped-read alignment with Bowtie 2. *Nat Methods* 2012; **9**: 357–359.
3. Albertsen M, Hugenholtz P, Skarshewski A, Nielsen KL, Tyson GW, Nielsen PH. Genome sequences of rare, uncultured bacteria obtained by differential coverage binning of multiple metagenomes. *Nat Biotechnol* 2013; **31**: 533–538.
4. Prjibelski A, Antipov D, Meleshko D, Lapidus A, Korobeynikov A. Using SPAdes de novo assembler. *Curr Protoc Bioinforma* 2020; **70**: e102.
5. Parks DH, Imelfort M, Skennerton CT, Hugenholtz P, Tyson GW. CheckM: assessing the quality of microbial genomes recovered from isolates, single cells, and metagenomes. *Genome Res* 2015; **25**: 1043–1055.
6. Lee I, Kim YO, Park S-C, Chun J. OrthoANI: An improved algorithm and software for calculating average nucleotide identity. *Int J Syst Evol Microbiol* 2016; **66**: 1100–1103.
7. Kuraku S, Zmasek CM, Nishimura O, Katoh K. aLeaves facilitates on-demand exploration of metazoan gene family trees on MAFFT sequence alignment server with enhanced interactivity. *Nucleic Acids Res* 2013; **41**: W22–W28.
8. Capella-Gutiérrez S, Silla-Martínez JM, Gabaldón T. trimAl: a tool for automated alignment trimming in large-scale phylogenetic analyses. *Bioinformatics* 2009; **25**: 1972–1973.
9. Stamatakis A. RAxML version 8: a tool for phylogenetic analysis and post-analysis of large phylogenies. *Bioinformatics* 2014; **30**: 1312–1313.
10. Tanabe AS. Kakusan4 and Aminosan: two programs for comparing nonpartitioned, proportional and separate models for combined molecular phylogenetic analyses of multilocus sequence data. *Mol Ecol Resour* 2011; **11**: 914–921.
11. Zhu W, Lomsadze A, Borodovsky M. *Ab initio* gene identification in metagenomic sequences. *Nucleic Acids Res* 2010; **38**: e132.
12. Kelley DR, Liu B, Delcher AL, Pop M, Salzberg SL. Gene prediction with Glimmer for metagenomic sequences augmented by classification and clustering. *Nucleic Acids Res* 2012; **40**: e9.
13. Lowe TM, Chan PP. tRNAscan-SE On-line: integrating search and context for analysis of transfer RNA genes. *Nucleic Acids Res* 2016; **44**: W54–W57.
14. Nawrocki EP, Eddy SR. Infernal 1.1: 100-fold faster RNA homology searches. *Bioinformatics* 2013; **29**: 2933–2935.
15. Krogh A, Larsson B, von Heijne G, Sonnhammer ELL. Predicting transmembrane protein topology with a hidden markov model: application to complete genomes. *J Mol Biol* 2001; **305**: 567–580.
16. Almagro Armenteros JJ, Tsirigos KD, Sønderby CK, Petersen TN, Winther O, Brunak S, et al. SignalP 5.0 improves signal peptide predictions using deep neural networks. *Nat Biotechnol* 2019; **37**: 420–423.
17. Yu NY, Wagner JR, Laird MR, Melli G, Rey S, Lo R, et al. PSORTb 3.0: improved protein subcellular localization prediction with refined localization

subcategories and predictive capabilities for all prokaryotes. *Bioinformatics* 2010; **26**: 1608–1615.

18. Yu C-S, Lin C-J, Hwang J-K. Predicting subcellular localization of proteins for Gram-negative bacteria by support vector machines based on *n*-peptide compositions. *Protein Sci* 2004; **13**: 1402–1406.
